# *Alyssum homolocarpum* seed oil (AHSO), containing natural alpha linolenic acid, stearic acid, myristic acid and β-sitosterol, increases proliferation and differentiation of neural stem cells in vitro

**DOI:** 10.1186/s12906-019-2518-4

**Published:** 2019-06-03

**Authors:** Reza Mahmoudi, Majid Ghareghani, Kazem Zibara, Maryam Tajali Ardakani, Yahya Jand, Hassan Azari, Jafar Nikbakht, Amir Ghanbari

**Affiliations:** 1 Cellular and Molecular Research Center, Yasuj University of Medical Sciences, Yasuj, Iran; 2 Medicinal Plants Research Center, Yasuj University of Medical Sciences, Yasuj, Iran; 3 CERVO Brain Research Center, Quebec City, QC G1J 2G3 Canada; 4 Laboratory of stem cells, PRASE, DSST and Biology department, Faculty of Sciences-I, Lebanese University, Beirut, Lebanon; 5 Department of Pharmacology, School of Medicine, Tehran University of Medical Sciences, Tehran, Iran; 6 Neural Stem Cell and Regenerative Neuroscience Laboratory, Department of Anatomical Sciences, Shiraz School of Medicine and Shiraz Stem Cell Institute, Shiraz University of Medical Sciences, Shiraz, Iran

**Keywords:** Embryonic neural stem cell, Alyssum homolocarpum, Alpha-linoleic acid, Stearic acid, Myristic acid

## Abstract

**Background:**

Embryonic neural stem cells (eNSCs) are immature precursors of the central nervous system (CNS), with self-renewal and multipotential differentiation capacities. These are regulated by endogenous and exogenous factors such as alpha-linolenic acid (ALA), a plant-based essential omega-3 polyunsaturated fatty acid.

**Methods:**

In this study, we investigated the effects of various concentrations of *Alyssum homolocarpum* seed oil (AHSO), containing natural ALA, stearic acid (SA), myristic acid (MA), and β-sitosterol, on proliferation and differentiation of eNSCs, in comparison to controls and to synthetic pure ALA.

**Results:**

Treatment with natural AHSO (25 to 75 μM), similar to synthetic ALA, caused a significant ~ 2-fold increase in eNCSs viability, in comparison to controls. To confirm this proliferative activity, treatment of NSCs with 50 or 75 μM AHSO resulted in a significant increase in mRNA levels of notch1, hes-1 and Ki-67and NICD protein expression, in comparison to controls. Moreover, AHSO administration significantly increased the differentiation of eNSCs toward astrocytes (GFAP+) and oligodendrocytes (MBP+) in a dose dependent manner and was more potent than ALA, at similar concentrations, in comparison to controls. Indeed, only high concentrations of 100 μM AHSO, but not ALA, caused a significant increase in the frequency of neurons (β-III Tubulin+).

**Conclusion:**

Our data demonstrated that AHSO, a rich source of ALA containing also other beneficial fatty acids, increased the proliferation and stimulated the differentiation of eNSCs. We suggest that AHSO’s effects are caused by β-sitosterol, SA and MA, present within this oil. AHSO could be used in diet to prevent neurodevelopmental syndromes, cognitive decline during aging, and various psychiatric disorders.

## Background

Dysregulation of fatty acid and phospholipid metabolism in the central nervous system (CNS) have been linked to a wide range of neurological, psychiatric, and developmental brain disorders [1]. Stimulation of neurogenesis is considered as an important tool to treat brain disorders, particularly neurodegenerative diseases [2].

Omega-3 family of polyunsaturated fatty acids (PUFAs), such as Eicosapentaenoic acid (EPA) and Docosahexaenoic acid (DHA), are essential for the developing brain and comprise approximately 8% of its weight [3]. Indeed, both EPA and DHA play critical roles in neuronal structure and function [4, 5]. Currently, the importance and function of other fatty acids (FAs) is under investigation. Recently, our team confirmed that alpha-linolenic acid (ALA), an omega-3 essential FA, causes an increase in proliferation and differentiation of cultured neural stem cell (NSCs) [6]. Previously, our team and others reported the proliferative activity of β-sitosterol, a phytosterol, on NSCs in vitro [7]. On the other hand, some studies reported the differentiative activity of stearic acid (SA) and myristic acid (MA) in the brain, two common saturated FAs with 18 and 14 carbons, respectively [8, 9]. One of the richest herbal sources for fatty acids is *Alyssum homolocarpum* seed oil (AHSO), which was shown to induce proliferation of NSCs, but not their differentiation [10]. Alyssum, a famous genus of Brassicaceae or mustard family, is native to the Middle East, especially Iran, Iraq and Pakistan, and comprises 100–170 related species. *Alyssum homolocarpum* plant is well known by Persian practitioners and folk healers and is traditionally known as Qodume Shirazi or Toodari [11–14].

The exact mechanism by which PUFAs exert their beneficial effects on neurogenesis has not been elucidated yet. We hypothesized that the presence of AHSO prior to differentiation is important to induce its activity on NSCs in vitro. The current study is designed to investigate the direct effect of AHSO on proliferation, but also differentiation, of NSCs and to compare its activities with a synthetic source of ALA.

## Methods

### Sample collection and plant identification

*Alyssum homolocarpum* seeds were collected from mountains of Shiraz city in Iran and authenticated by taxonomist Ms. Sedigheh Khademyan, similar to our previously published paper [10]. The voucher specimen was preserved with the code PM-53 at the Department of Pharmacognosy, School of Pharmacy, Shiraz University of Medical Sciences for any further reference.

### Oil components

The fatty acid profile of *Alyssum homolocarpum* contains 90% alpha linolenic acid (ALA), 2.4% stearic acid (SA), 1.8% myristic acid (MA), 1.02% arachidic acid (AA), 3.3 mg/g β- sitosterol and 5% of other fatty acids (tetradecanoic acid, 12-methyl, 9-hexadecenoic acid, 11-hexadecenoic acid, hexadecanoic acid, heptadecanoic acid, 11,14,17-eicosatrienoic acid, docosanoic acid), as previously determined [10].

### Animals

Mice were purchased from Razi Institute (Shiraz, Iran). They were maintained and housed at the Animal Breeding Center under controlled temperature and humidity conditions as well as pathogen-free environment. All experimental procedures in this study were approved by the Institutional Animal Care and Use Committee (IACUC) of Yasuj University of Medical Science (Permission number; IR.YUMS.REC.1395.2). Animal procedures were carried out in accordance with the guidelines of the Iranian Agriculture Ministry, which conforms to the international laws and policies (NIH Guide for the Care and Use of Laboratory Animals, NIH Publication No. 85–23, 1985, revised 1996). Mice were orally administered with 0.5 g/kg/day of AHSO during the total period of their gestation until 14 days. Pregnant BALB/c mice (25–30 g) at E14 were used to generate neural stem cells (NSCs). To prevent mice suffering, pain and distress, pregnant mice were euthanized by cervical dislocation under ether narcotization. All efforts were made to minimize animal suffering and to reduce the number of animals used.

### Isolation and expansion of eNSCs

Primary cultures of embryonic NSCs were prepared as described previously [15]. Briefly, brains were removed under sterile conditions and cerebral cortices of E14 mice were micro-dissected then disrupted into single cells by repeated pipetting. Cells were then plated, at a density of 1 × 10^5^ cells/mL, in a T-25 flask. The medium for cell culture consisted of DMEM/F-12 (Invitrogen) supplied with 20 ng/ml each of epidermal growth factor and basic fibroblast growth factor (both from Peprotech), 2% B27 supplements (Invitrogen), and 2 μg/mL heparin (Sigma-Aldrich, USA). Cell cultures were kept at 37 °C in a humidified atmosphere with 5% CO_2_ for 5–6 days [15]. When neurospheres were obtained, they were passaged with 0.05% trypsin/EDTA (Sigma-Aldrich).

### Cell viability assay

A colorimetric cell viability assay was performed to determine cell densities using MTT [3-(4,5-dimethylthiazol-2-yl)-2,5-diphenyltetrazolium bromide]. Briefly, cells derived from the primary cultures of neurospheres were dissociated and then seeded on 96-well plates at a density of 5000 cells. Various concentrations of synthetic ALA or natural AHSO (25, 50, 75, 100, and 200 μM) were then used to treat the seeded cells for 48 h. Cells were maintained at 37 °C in a humidified atmosphere of 5% CO_2_, as previously described [6]. After cell treatment, ALA and AHSO-containing media were removed and wells were gently washed twice with 1× PBS followed by the addition of 200 μl of fresh medium, containing 0.5 mg/ml of MTT, into each well. Plates were then incubated at 37 °C for 4 h. Cells were then disrupted in a solubilizing solution containing dimethyl sulfoxide (DMSO) and ethanol at a 1:1 ratio. The formazan product was quantified at 460 nm absorbance in an ELISA microplate reader. A total of five independent experiments were conducted (*n* = 5).

### Determination of neurosphere frequency using Neurosphere assay

Cells derived from dissociated neurospheres at passage 2 were seeded in 96-well plates at a density of 5000 cells in the neurosphere medium. Cells were then treated with different concentrations of synthetic ALA or natural AHSO (25, 50, 75, 100, and 200 μM). After 5 days, neurospheres with dimensions bigger than or equal to 50 μm in diameter were counted using an Olympus inverted light microscope. Results were expressed as the percent neurosphere-forming frequency per well reported to the total number of cells that were initially plated. At the same time and through a systematic random sampling method, a total of 4–5 images were captured for each well, using an Olympus digital camera. A total of 10 replicates were carried out for each concentration and the mean of all wells (*n* = 10) was considered as the sphere forming frequency for each condition.

### Real-time PCR

Using QIAGEN RNeasy Kit (Qiagen, Japan), total RNA from NSCs was extracted followed by cDNA synthesis using random primers (Applied Biosystems, USA) and the High-Capacity cDNA Reverse Transcription kit. Quantitative real-time PCR (qPCR) was then performed using the StepOne Real-Time PCR system (Applied Biosystems) and with RealQ Plus 2x Master Mix Green (Ampliqon, Denmark), according to manufacturer’s protocols. The primer sequences used were: Notch1, F: TGGTTCAGGGCGGTGCTCA and R: CAGACACCTGCTTCCCAAAAGG; Hes1, F: TTCCTCCCATTGGCTGAAAG and R: CCAGCTCCAGATCCAGTGTGAT; Ki-67, F: GAGCAGTTACAGGGAACCGAAG and R: CCTACTTTGGGTGAAGAGGCTG; GAPDH, F: ATCTTCTTGTGCAGTGCCAGC and R: CCTTGACTGTGCCGTTGAACT. The specificity of PCR products was confirmed using melting curve analysis approach (data not shown). The PCR conditions were as follows: initial denaturation at 95 °C for 15 min, then 35 cycles of denaturation at 95 °C for 15 s, annealing at 57 °C for 30 s, and extension at 72 °C for 30 s. The Comparative CT (ΔΔCT) method was used to determine the relative changes in gene expression levels. GAPDH was used as an internal control and all reactions were performed in triplicate.

### Western blotting

Cells were homogenized on ice and lysed in a lysis buffer containing 50 mM Tris–HCl (pH 7.5), 150 mM NaCl, 1 mM PMSF, 100 mg/ml leupeptin, 1% Nonidet P40, 0.5% deoxycholic acid and 0.1% SDS. A colorimetric protein assay kit (Bio-Rad, USA) was then used to measure proteins. A total of 40 μg total proteins were resolved on 8–15% SDS-PAGE before transferring them onto a nitrocellulose membrane. Blocking the membranes was performed using 5% skim milk solution for 1 h followed by their incubation with a primary antibody against β-actin (1:1000, Santa Cruz Biotechnology Inc., USA) or NICD (1:500, Abcam, USA) for an overnight on shaker at 4 °C. After washing steps, species-appropriate horseradish peroxidase-conjugated secondary antibodies were incubated at room temperature. Immunoreactive proteins were then detected using an enhanced chemiluminescence Western blotting detection system. Protein bands were then scanned by densitometry using MyImage (SLB, Seoul, Korea) and quantified by an image analysis software (LabWorks Software Version 3.0, UVP Inc., USA).

### Neural stem cells differentiation

Neurospheres at passage 2 were dissociated into single cells using 0.05% trypsin-EDTA (Gibco, USA). Cells were then seeded at a density of 1 × 10^6^ cells/well on 2-well plates (Sigma-Aldrich) coated with poly-L-ornithine (15 mg/mL), using N2 medium without bFGF and EGF. Experimental groups contained cells which were treated with synthetic ALA (25, 50, and 100 μM) or AHSO (25, 50, and 100 μM), dissolved in N2 medium and containing 1% bovine serum albumin (BSA, Gibco, USA) at 0.01% final concentration. As a positive control, PDGF (30 ng/ml), an oligodendrocyte promoting factor, was used. The culture medium was changed every other day for a total of 5 days.

### Immunofluorescence analysis

Differentiated cultures were then used for analysis by immunofluorescence (IF). Cells were fixed for 20 min in 2% paraformaldehyde (PFA) in 12-well plates followed by washing with PBS. Thereafter, cells were permeabilized for 20 min using 20% tween and then blocked for 20 min in PBS, 2% triton, 5% horse serum (PBSTS). Thereafter, cells were incubated overnight with monoclonal antibodies against myelin basic protein (MBP), glial fibrillary acidic protein (GFAP), and β-III tubulin (Sigma Aldrich, USA) at 1:500, 1:800 and 1:700 dilutions, respectively. Following several washes in PBS, wells were incubated for 1 h with Alexa Fluor 488 or 568 (1:500 dilution) conjugated anti-mouse or anti-rabbit secondary antibodies, respectively. Representative pictures of each well (10–12 fields/well) were taken using a fluorescent microscope (Olympus IX-71) equipped with a Canon EOS digital camera. Cells were then counted, and data presented as percent positive cells per treatment condition.

### Statistical analysis

Results are presented as the mean with error bars indicating the standard error of the mean (Mean ± SEM). Statistical analysis was performed using GraphPad Prism (Version 6.01, San Diego, CA, USA) software. Data analysis was performed with Ordinary one-way ANOVA followed by Tukey’s multiple comparison test, with a single pooled variance. Significance is indicated by ∗, *p* < 0.05; ∗∗, *p* < 0.01; ∗∗∗, *p* < 0.001 and ****, *p* < 0.0001.

## Results

### Proliferation of embryonic NSCs is altered by ALA and AHSO

**Fig. 1:**
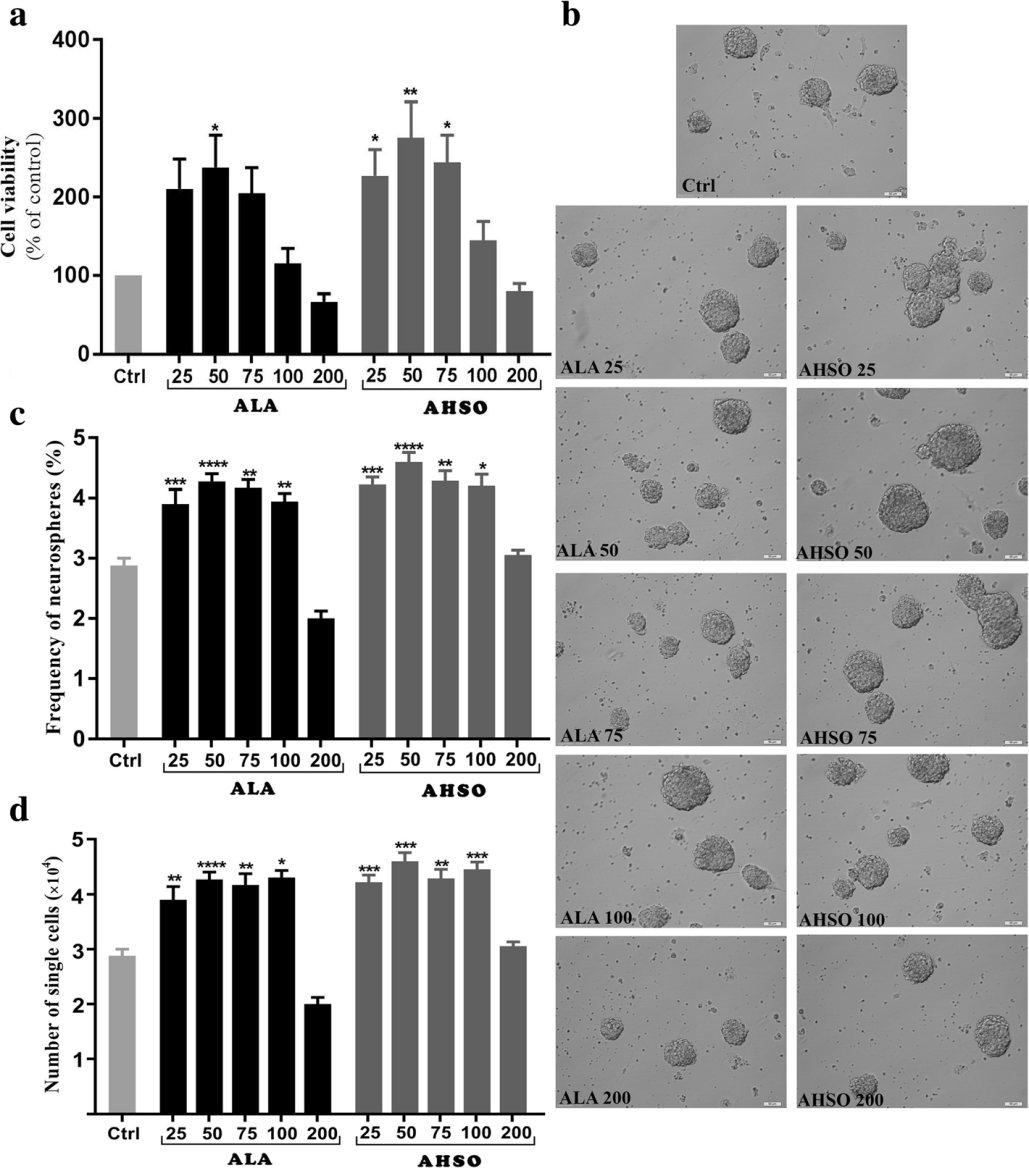
Effects of ALA and AHSO on viability and neurosphere formation of eNSCs in vitro. Cells were exposed to different concentrations of synthetic ALA or natural AHSO (25, 50, 75, 100 and 200 μM) for 48 h, then subjected to analysis. **a** Cell viability assessed by MTT assay. **b** Representative captures of neurospheres in different groups. Scale bars: 100 휇m. **c** eNSCs at a density of 500 cells/well were exposed to different concentrations of ALA or AHSO for 5 days. Neurosphere formation was monitored under a microscope and the frequency of neurospheres with a diameter > 50 μm were analyzed. **d** Cell counts obtained from neurospheres. A one-way analysis of variance (ANOVA) following Tukey post-hoc test was performed to compare the mean values. Data were shown as mean ± SEM. *, *p* < 0 .05, **, p < 0 .01, ***, *p* < 0.001 and ****, *p* < 0.0001 versus control

Safe concentrations of ALA and AHSO were first determined since they may exert cytotoxic effects on eNSCs. Therefore, eNSCs were incubated with either synthetic ALA or AHSO, containing natural ALA, at similar concentrations ranging from 25 to 200 μM. Results showed that concentrations of 25, 50, and 75 μM of ALA caused ~ 2-fold increase in eNCSs viability, in comparison to controls, as revealed by MTT assay (Fig. 1a). However, only 50 μM was significant while 200 μM was toxic (Fig. 1a). On the other hand, AHSO treatment showed a similar trend, but stronger, since concentrations of 25, 50, and 75 μM of ALA caused a significant increase in eNCSs cell viability (Fig. 1a). Based on these data, concentrations of ≤100 μM of ALA or AHSO were used in further experiments.

The effects of ALA and AHSO were then examined on the proliferation of NSCs using the neurosphere assay, which reflects the self-renewal potential of NSCs when they are plated at a very low density. In this study, NSCs formed neurospheres of various sizes ranging between 50 휇m to > 100 휇m in diameter (Fig. 1b). Results showed that AHSO concentrations ranging between 25 and 100 μM, but also similar concentrations of ALA, caused a significant increase in neurosphere frequency, in comparison to controls (*n* = 10 replicates for each concentration), with a maximum stimulatory effect of AHSO or ALA at 50 μM concentrations (Fig. 1c).

The actual cell number obtained from neurospheres was then calculated. Consistent with neurosphere assay data, cell counts showed similar results at various concentrations of ALA and AHSO, with a maximum stimulatory effect at 50 μM (Fig. 1d).

### ALA and AHSO affects bHLH transcription factors and Ki-67 proliferative marker

**Fig. 2:**
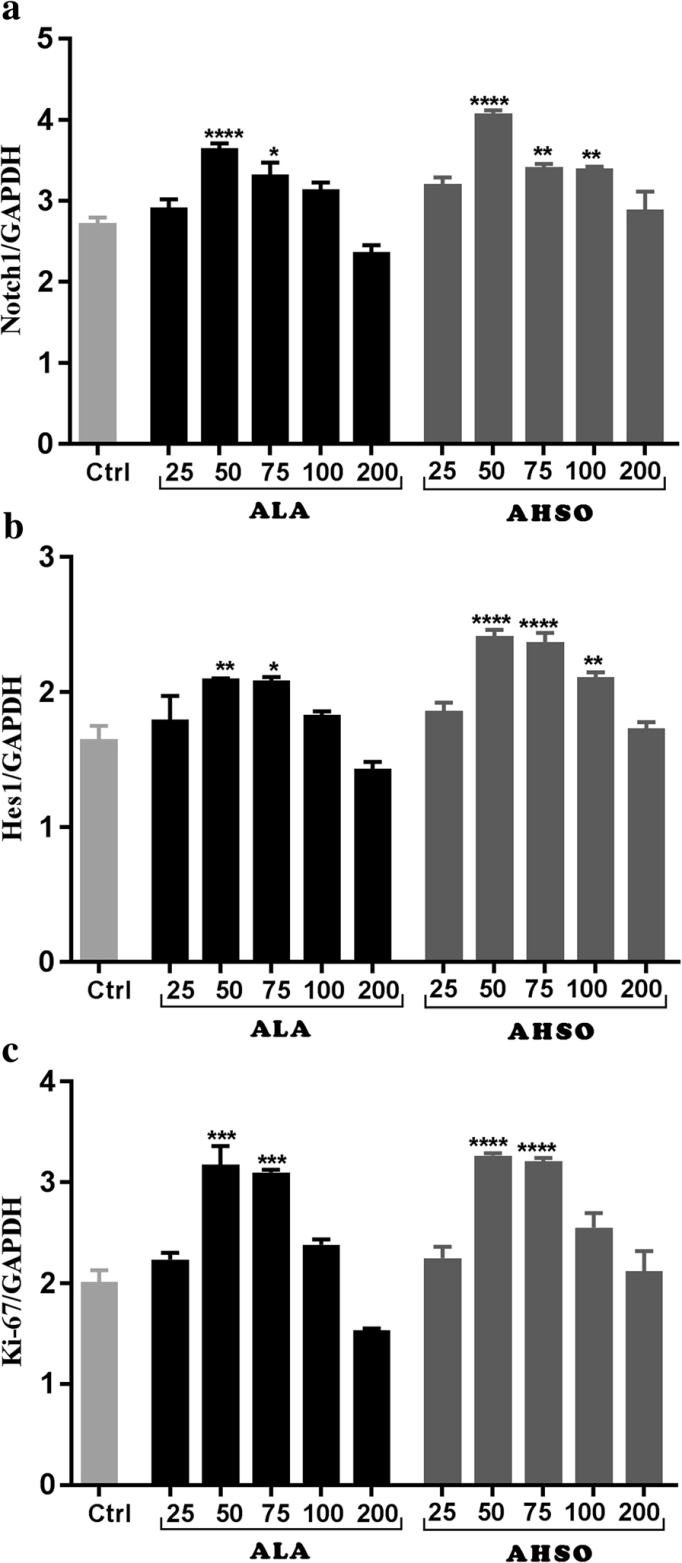
Effects of ALA and AHSO on Notch1, Hes1 and Ki-67. Cells were exposed to different concentrations of ALA or AHSO (25, 50, 75, 100, and 200 μM) for 5 days. Total RNA was prepared from each culture, cDNA synthesized and subjected to real-time PCR, using specific primers for Notch1 (**a**), Hes1 (**b**), or Ki-67 (**c**). GAPDH was used as an internal control. The values are expressed as the mean ± SEM. A one-way analysis of variance (ANOVA) following Tukey post-hoc test was performed to compare the mean values. *, *p* < 0.05; **, *p* < 0.01; and ****, p < 0.0001 were considered significant

Notch1 and hes1 are well-known bHLH transcription factors involved in promoting the stemness of NSCs. In order to understand the mechanism by which ALA and AHSO increase the proliferation of NSCs, notch1 and hes1 transcription factors were examined in addition to Ki-67 proliferation marker. Real-Time PCR results showed that ALA at concentrations of 50 and 75 μM increased significantly (****, *p* < 0.0001 and *, *p* < 0.05, respectively) notch1 expression, in comparison to controls (Fig. 2a). Similarly, treatment of NSCs with 50 or 75 μM concentrations of AHSO resulted in a significant increase (****, p < 0.0001 and **, *p* < 0.01, respectively) in mRNA expression levels of notch1, in comparison to controls (Fig. 2a). In the same manner, expression levels of hes1 **(**Fig. 2b**)** and Ki-67 **(**Fig. 2c**)** transcripts were significantly enhanced at concentrations of 50 and 75 μM of AHSO or ALA, in comparison to controls (****, *p* < 0.0001).

### Effect of ALA and AHSO on NICD protein levels

**Fig. 3:**
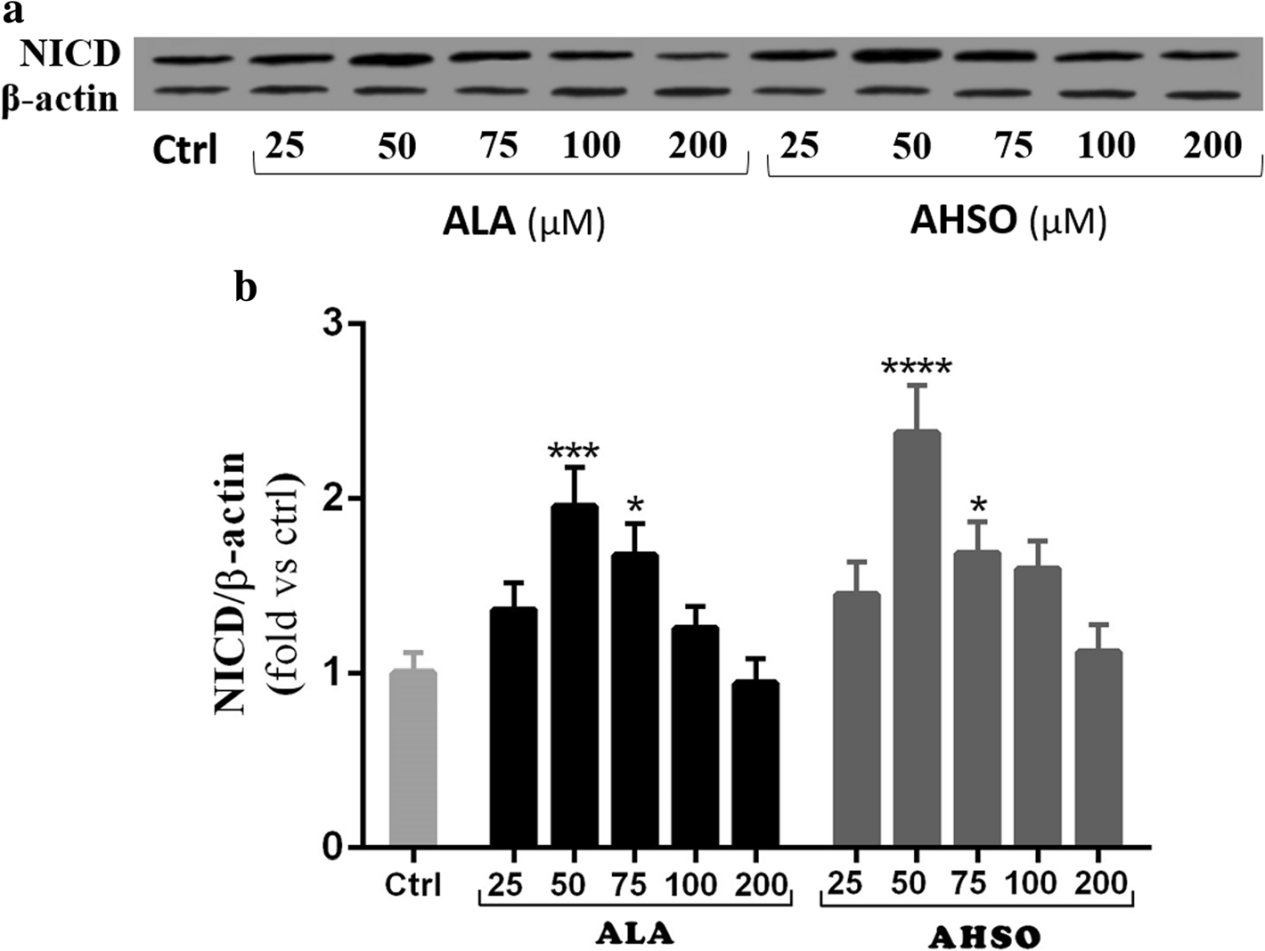
Effect of ALA and AHSO on NICD protein expression levels. **a** Representative western blot showing NICD expressions. **b** Quantification of NICD expressions in all groups. β-actin was used as an internal control for normalization. Values are expressed as the Mean ± SEM. Each group included 3 replicates (*n* = 3). Statistical analysis was performed by one-way analysis of variance followed by Tukey’s test. Significance is indicated by *p < 0.05, ***p < 0.001 and ****p < 0.0001

The expression of hes1 is regulated by Notch protein which in turn is cleaved by γ-secretase responsible for releasing Notch intracellular domain (NICD). It has been demonstrated that NICD moves into the nucleus and induces hes1 expression that inhibits the differentiation of NSCs [16]. Consistent with real time-PCR results, western blotting showed that treatment of NSCs with 50 or 75 μM concentrations of ALA caused a significant increase in NICD protein expression, in comparison to controls **(**Fig. 3a and b**)**. Equally, similar AHSO concentrations showed a further significant increase in NICD protein expression levels, in comparison to controls (~ 2.4- vs ~ 1.8-fold; ****, p < 0.0001 vs **, *p* < 0.01, respectively) (Fig. 3a and b). These data are in accordance with transcriptional expression results of notch1 and hes1.

### ALA and AHSO treatments affect the differentiation of embryonic NSCs

**Fig. 4:**
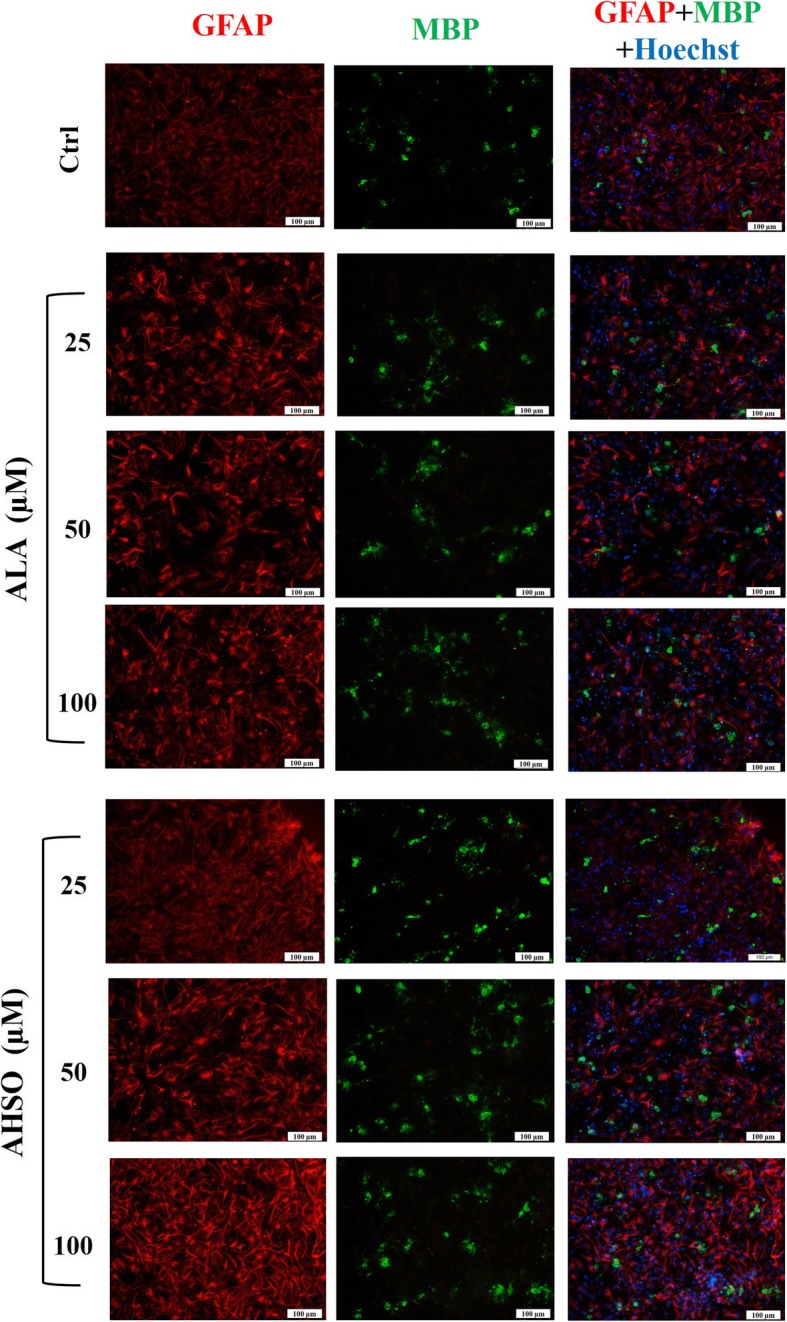
The effects of ALA and AHSO on differentiation of cultured eNSCs toward astrocyte and oligodendrocyte cells. eNSCs dissociated from primary neurospheres were differentiated in 1% FBS-containing medium lacking EGF. Five days after treatment with various concentrations of ALA or AHSO, cells were fixed with 4% PFA and processed by immunocytochemistry. Representative images of double immunofluorescence for astrocytes (GFAP, red) and oligodendrocytes (MBP, green). The blue color indicates nuclear staining (Hoechst). Scale bar = 100 nm

**Fig. 5:**
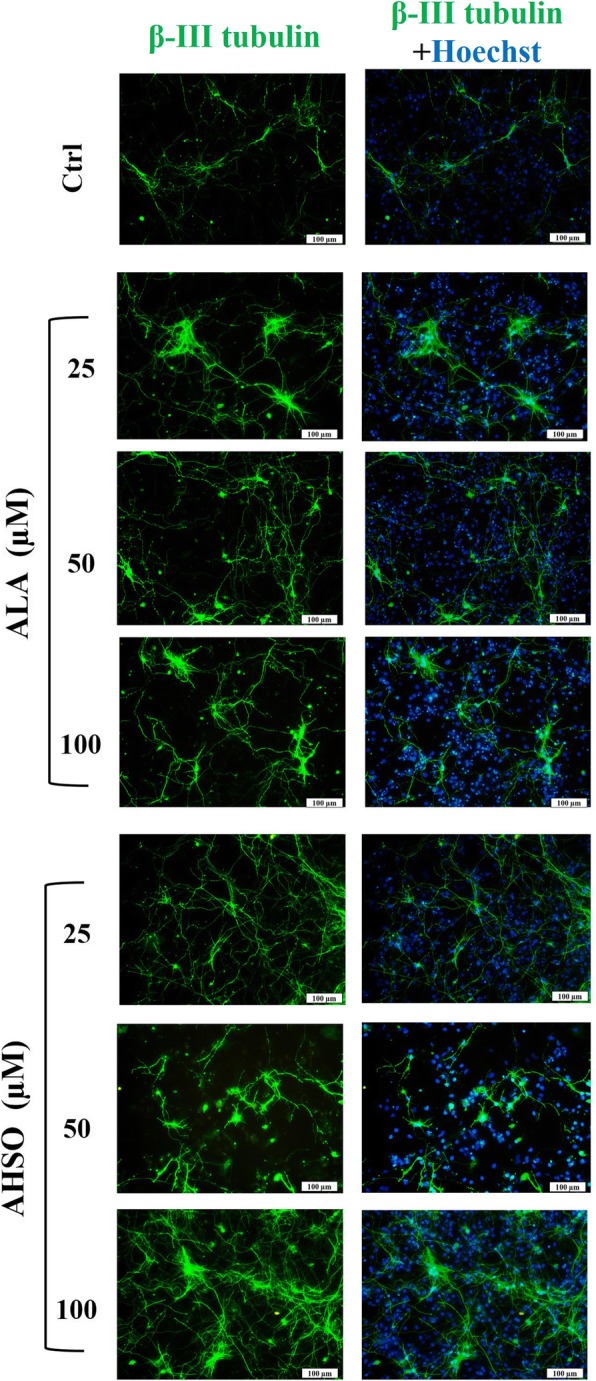
The effects of ALA and AHSO on differentiation of cultured eNSCs toward neuron. Representative immunofluorescence images for neurons (β-III tubulin, green). The blue color indicates nuclear staining (Hoechst). Scale bar = 100 nm

**Fig. 6:**
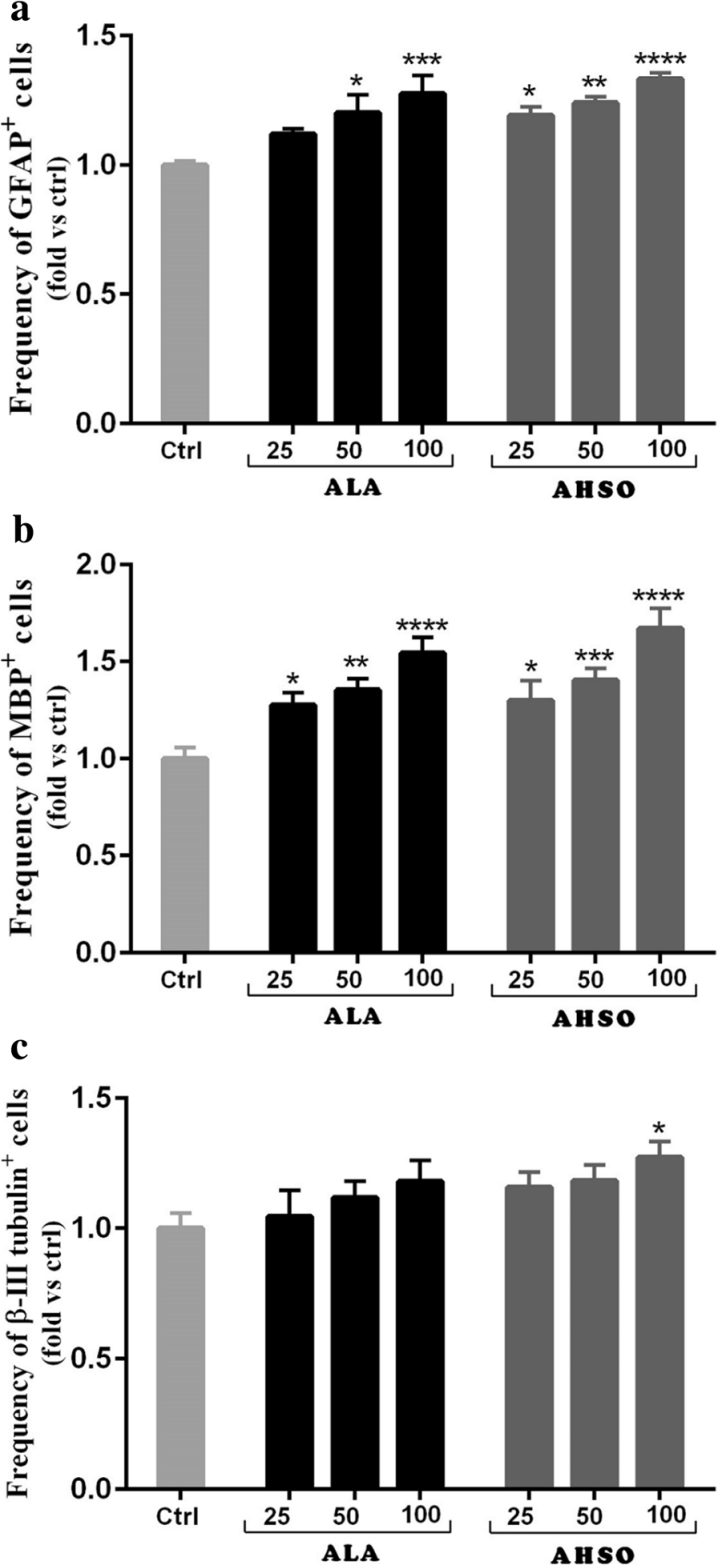
Quantitative analysis of immunofluorescence staining. **a** and **b** ALA and AHSO significantly enhance the frequency of astrocytes (GFAP) and oligodendrocytes (MBP). **c** ALA did not affect the differentiative activity of eNSCs into neurons, however, higher concentrations of AHSO (100 μM) significantly increased the frequency of β-III tubulin positive cells, in comparison to controls. The asterisks indicate statistical significance with respect to controls (*, p < 0.05; **, p < 0.01; ***, p < 0.001; and ****, p < 0.0001; one-way ANOVA followed by Tukey multiple test). Data are presented as means ± SEM. *n* = 15

To evaluate the effect of ALA and AHSO on eNSC differentiation, cells were differentiated for 5 days and then immuno-stained with markers of mature astrocytes (glial fibrillary acidic protein, GFAP; Fig. 4), oligodendrocytes (myelin basic protein, MBP; Fig. 4), and neurons (β-III tubulin; Fig. 5). Results showed that ALA treatment significantly increased the frequency of GFAP and MBP positive cells in a dose dependent manner, in comparison to controls **(**Fig. 4 and Fig. 6**)**. At similar concentrations, AHSO administration significantly increased, in a dose dependent manner and more potently than ALA, the differentiation of eNSCs toward GFAP and MBP positive cells **(**Fig. 6a and b**).** Interestingly, AHSO, but not ALA, also significantly (*p < 0.05) increased the frequency of GFAP positive cells at 25 μM, in comparison to controls **(**Fig. 4 and Fig. 6a**)**.

On the other hand, low or high concentrations of ALA had no significant increase on the differentiation of NSCs toward neurons. However, high concentrations of 100 μM AHSO caused a significant (*p < 0.05) increase in the frequency of β-III Tubulin positive cells, in comparison to controls **(**Fig. 5 and Fig. 6c**)**.

Taken together, these observations suggest that synthetic ALA, but also natural AHSO, play a major role in the differentiation of eNSCs toward astrocytes and oligodendrocytes.

## Discussion

*Alyssum homolocarpum* seed oil (AHSO) contains alpha linolenic acid (ALA), stearic acid (SA), myristic acid (MA), and β-sitosterol. In the present study and for the first time, we give evidence of the beneficial effects of AHSO on the proliferation and differentiation of cultured NSCs. Several lines of evidence support this statement: (1) A significant increase of ~ 2-fold in eNCSs viability was obtained using natural AHSO at 25, 50, and 75 μM whereas synthetic ALA was significant at 50 μM only. (2) Concentrations of 25 to 100 μM of AHSO, similar to ALA, caused a significant increase in the neurosphere frequency and their cell counts, with a maximum stimulatory effect at 50 μM. (3) Concentrations of 50 or 75 μM of AHSO, but also ALA, significantly increased the transcript levels of notch1, hes1 and Ki-67. (4) AHSO caused a significant increase in NICD protein expression. (5) AHSO, more potently than ALA, significantly increased the differentiation of eNSCs toward GFAP and MBP positive cells, in a dose dependent manner. (6) High concentrations of 100 μM AHSO, but not ALA, significantly increased the frequency of β-III Tubulin positive cells.

We have previously revealed that synthetic ALA increases the proliferation capacity of eNSCs at concentrations ranging between 25 and 100 μM, with an optimal concentration of 50 μM [6]. In this study, AHSO showed an increase in eNSCs proliferation, similarly to synthetic ALA at comparable concentrations. Indeed, AHSO at a concentration of 50 μM was found to be the optimal dose to induce proliferation, as confirmed by MTT and neurosphere forming assay. It is worth noting that AHSO contains natural ALA, along with SA, MA, and β-sitosterol.

At the molecular level, several signaling pathways, among which Notch1 signaling pathway, have been implicated in the mechanisms of proliferation and differentiation of NSCs and in regulating CNS development [17]. For instance, Notch1 and hes1 have been reported to promote cell proliferation and self-renewal of NSCs [18, 19]. In addition, upon activation of Notch, NICD was shown to be released from the membrane, translocating to the nucleus where it induces the expression of transcriptional repressor genes such as hes1, inhibiting neuronal differentiation. Hence, the activation of Notch1 signaling could play a role in maintaining NSCs in undifferentiated states while increasing the stemness of NSCs [20–23]. Therefore, we investigated the gene expression of basic helix–loop–helix (bHLH) transcription factors including Notch1, hes1 as well as Ki-67 proliferation marker in order to further confirm the proliferative effects of ALA and AHSO. Our data confirmed the stimulatory effect of ALA and AHSO, at 50 and 75 μM concentrations, on the stemness and proliferation of NSCs. Moreover, ALA and AHSO stimulated NICD protein expression at these concentrations.

Given that NSCs are heterogeneous populations, not only they comprise bona fide stem cells but also progenitor cells of different neural cell lineages including oligodendroglial, astrocytic and neuronal progenitors [24]. In this study, when NSCs were cultured in a proliferation medium in the presence of growth factors, AHSO was shown to increase the stemness property and proliferation, via Notch 1 signaling. Interestingly, when NSCs were plated in a differentiation medium in the absence of growth factors, an overall increase of differentiation in the three neural lineages was observed. This could be due to an overall increase of cell viability in presence of AHSO, as shown in this study.

The fatty acid profile of AHSO was previously analyzed and found to contain 89.7% ALA, 2.4% SA, 1.8% MA, 1.02% Arachidic acid (AA), 3.3 mg/g β- sitosterol and 5% of other fatty acids (Tetradecanoic acid, 12-methyl, 9-Hexadecenoic acid, 11-Hexadecenoic acid, Hexadecanoic acid, Heptadecanoic acid, 11,14,17-Eicosatrienoic acid, Docosanoic acid) [10]. Indeed, AHSO contains natural ALA to nearly similar concentrations to synthetic ALA. In addition, AHSO also contains other fatty acids. For instance, one of these FAs is β-sitosterol which we recently found to significantly enhance the proliferation of NSCs, however, it couldn’t affect neural differentiation [25]. Similarly, it has been shown that β-sitosterol-D-glucoside is an efficient and inexpensive growth factor, which could promote NSC proliferation [7]. To our knowledge, these are the only two studies which investigated the role of β-sitosterol on NSCs, which could be considered as a potential agent for enhancing the proliferative activity of ALA, as observed in AHSO treated eNSCs.

Stearic acid (SA) is the other main PUFA in AHSO at a percentage of 2.4%. Long ago, it has been reported that injected SA is uptaken by the brain and is further incorporated into membrane lipids, especially myelin, or metabolized into longer chains lipids [8]. This suggests that SA could be a beneficial agent for neural differentiation, particularly toward myelin forming oligodendrocytes. In this study, both ALA and AHSO, at concentrations ranging between 25 to 100 μM, increased the differentiative activity of eNSCs toward GFAP+ astrocytes and MBP+ oligodendrocytes. However, concentrations of 25 or 100 μM of AHSO, but not ALA, could increase neural differentiation toward astrocytes or β-III Tubulin-expressing neurons, respectively. It’s important to note that AHSO was always found to be more potent than ALA, at similar concentrations. This suggests that SA and MA, the two main PUFAs, could strengthen the differentiative capacity of ALA in AHSO-treated eNSCs.

Myristic acid (MA) has been shown to enhance ALA tissue storage and to increase docosahexaenoic acid (DHA) and Arachidonic acid (AA) concentrations in the brain of rats [9]. Also, it has been suggested that MA could be an activator of ALA conversion to DHA since it increases the activity of delta 6-desaturase in a dose dependent manner [26]. In addition, several studies reported that diets containing MA enhanced liver and plasma concentrations of eicosapentaenoic acid (EPA) and DHA [27–29]. Furthermore, it was shown that ~ 8% of dietary ALA is converted to EPA while 0–4% is converted to DHA [30]. Importantly, conversion of ALA into DHA was shown to promote eNSC maintenance since desaturases and elongases, required in ALA metabolism, are expressed in eNSCs [31]. In this study, high concentrations of ALA ensured adequate concentrations of its metabolites including DHA since there are limitations in converting ALA to DHA due to enzyme deficiency of fatty acid delta 6-desaturase [32].

DHA and AA have been previously reported to affect neurogenesis [33–35]. For instance, combination treatment of 40 μM each of DHA and AA resulted in a neuron-like cellular fatty acid composition and increased the population of neurons in human mesenchymal stem cells (MSCs) [33]. Moreover, an in vitro and in vivo study on fat-1 cells and DHA-treated mice, respectively, showed increased levels of neurogenesis leading to improvements in spatial learning [36]. In accordance, our data revealed that AHSO at 100 μM concentration, but not ALA, significantly increased the frequency of neurons. Taken together, we suggest that the higher concentrations of MA in AHSO could be the main agent for stimulation of neurogenesis. Importantly, although the conversion rate of ALA to DHA is low, the presence of a nutrient such as MA could help in reaching this goal by modulating both the level of cellular ALA as well as its conversion to DHA, as previously reported [9].

## Conclusion

Overall, our study suggests that diets containing high concentrations of natural ALA with other fatty acids such as β-sitosterol, stearic acid, and myristic acid could be an efficient strategy to strengthen the proliferative and differentiative activities of ALA, particularly for induction of neurogenesis. Further studies are needed to determine the optimal dose of these fatty acids for gliogenesis, oligodendrogenesis, or neurogenesis. *Alyssum homolocarpum* seed oil is a herbal source with various fatty acids, which could be essential in therapeutic interventions. This oil could be used to control neurodevelopmental syndromes, cognitive decline during aging, and various psychiatric disorders.
